# Spatial Tuning of Light–Matter Interaction via Strain-Gradient-Induced Polarization in Freestanding Wrinkled 2D Materials

**DOI:** 10.1021/acs.nanolett.3c02550

**Published:** 2023-10-05

**Authors:** Chullhee Cho, Zhichao Zhang, Jin Myung Kim, Peiwen J. Ma, Md Farhadul Haque, Peter Snapp, SungWoo Nam

**Affiliations:** † Department of Mechanical Science and Engineering, University of Illinois Urbana−Champaign, Urbana, Illinois 61801, United States; ‡ Cryogenics and Fluids Branch, NASA Goddard Space Flight Center, Greenbelt, Maryland 20771, United States; § Department of Mechanical and Aerospace Engineering, University of California, Irvine, Irvine, California 92697, United States; ∥ Detectors Systems Branch, NASA Goddard Space Flight Center, Greenbelt, Maryland 20771, United States; ⊥ Department of Materials Science Engineering, University of California, Irvine, Irvine, California 92697, United States

**Keywords:** strain engineering, freestanding wrinkles, TMDCs, photoinduced force, flexoelectricity

## Abstract

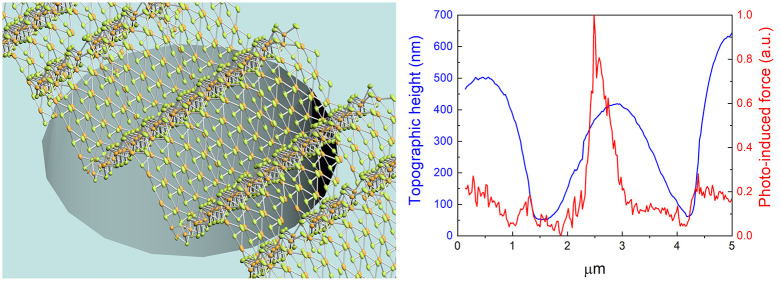

To date, controlled deformation of two-dimensional (2D) materials has been extensively demonstrated with substrate-supported structures. However, interfacial effects arising from these supporting materials may suppress or alter the unique behavior of the deformed 2D materials. To address interfacial effects, we report, for the first time, the formation of a micrometer-scale freestanding wrinkled structure of 2D material without any encapsulation layers where we observed the enhanced light–matter interactions with a spatial modulation. Freestanding wrinkled monolayer WSe_2_ exhibited about a 330% enhancement relative to supported wrinkled WSe_2_ quantified through photoinduced force microscopy. Spatial modulation and enhancement of light interaction in the freestanding wrinkled structures are attributed to the enhanced strain-gradient effect (i.e., out-of-plane polarization) enabled by removing the constraining support and proximate dielectrics. Our findings offer an additional degree of freedom to modulate the out-of-plane polarization and enhance the out-of-plane light–matter interaction in 2D materials.

Strain engineering is an attractive strategy to design and modulate material properties, via lattice deformation, to improve modern micro- and nanoelectronics device performance. Recently, two-dimensional (2D) materials, in particular 2D semiconducting transition metal dichalcogenides (TMDCs), have gained significant attention as fundamental building blocks for strain engineered devices including photovoltaics, optoelectronics, and quantum emitters.^1−5^ This is because few-atom-thick 2D TMDCs exhibit exceptional mechanical properties as well as quantum confinement allowing for precise tuning of optical properties. Furthermore, mechanical strain can strongly perturb the electronic band structures of 2D TMDCs, drastically modulating their electronic and optical characteristics,^3^ offering an effective strategy to achieve advanced strain-tunable optoelectronics.^6,7^

However, due to the challenge of deforming freestanding 2D materials without fracture,^8^ strain engineering of 2D materials has been demonstrated almost exclusively on substrate-supported 2D materials, where the 2D materials are in contact with an underlying deformable substrate.^2,3^ Unfortunately, the presence of an underlying substrate results in interfacial effects, including inefficient strain transfer resulting from Young’s modulus mismatch^9,10^ and surrounding dielectric effects which can suppress the luminescence efficiency of monolayer TMDCs.^5,9,11−13^ While several strategies have been developed to mitigate these substrate effects including the use of van der Waals substrates such as mica or hBN to mitigate the substrate doping effects^14^ or tuning substrate thickness to engineer light outcoupling in 2D TMDCs via light capture,^15^ the fundamental limitations arising from mechanical modulus mismatch and the dielectric screening from substrates still persist in the 2D TMDCs, limiting investigations of fundamental physics and the strain tunability of 2D materials.

Limited studies that have attempted to study deformed freestanding materials have relied on observations of uncontrolled or transfer-induced nanoscale ripples^16−18^ to investigate strain effects on 2D materials. Generation of ripples in suspended graphene has been reported by using strain-driven wrinkling resulting from thermal stress mismatch between graphene and the bottom silicon/silicon oxide substrate.^19^ However, due to the nanoscale dimension (sub-10 nm) of the ripples generated,^16−19^ the resultant strain effects assessed were mostly limited to mechanical properties (e.g., stiffness modulation).

Here, we introduce a novel strategy to create micrometer-scale freestanding wrinkled 2D TMDCs via a combination of a droplets-assisted transfer and mechanical prestrain release mechanism on a patterned, stretchable elastomeric substrate. By eliminating substrate constraints, we can concentrate the generated strain-gradient effects within the 2D TMDCs, thereby substantially enhancing the out-of-plane polarization (i.e., flexoelectric polarization). The enhanced light–matter interaction with the enhanced out-of-plane polarization in the freestanding wrinkled structure of 2D TMDCs is measured via near-field optical characterization using photoinduced force microscopy (PiFM) and compared with the optical responses from the substrate-supported wrinkled structures.

First, to create a flat freestanding 2D structure, a target TMDC monolayer was prepared via direct mechanical exfoliation on a polymer double layer consisting of poly(acrylic acid) (PAA) over a poly(methyl methacrylate) (PMMA) spin-coated on a SiO_2_/Si wafer (Figure 1a-i). The 2D material/polymer stack was then gently pressed onto a polydimethylsiloxane (PDMS) substrate (Figure 1a-iv), which had been embossed with circular cavities having 4–5 μm radius via a molding over a photolithographically patterned template consisting of microsized columns. Next, via our droplets-assisted transfer technique, a few droplets of a deionized (DI) water and methanol (1:1 ratio) mixture were applied with a micropipet at the interface between the PDMS and the water-soluble PAA layer (Figure 1a-v). Mixing DI water with methanol solvent reduces the surface tension of DI water approximately by half, allowing for much gentle removal of PAA layer without damaging the target 2D TMDC layer. After the sacrificial PAA layer was fully removed, the prepatterned PDMS stamp with 2D material on top was gently retracted (Figure 1a-vi). Using this technique, we successfully created freestanding 2D materials without any additional encapsulation layers (Figures 1b and S1).

**Figure 1:**
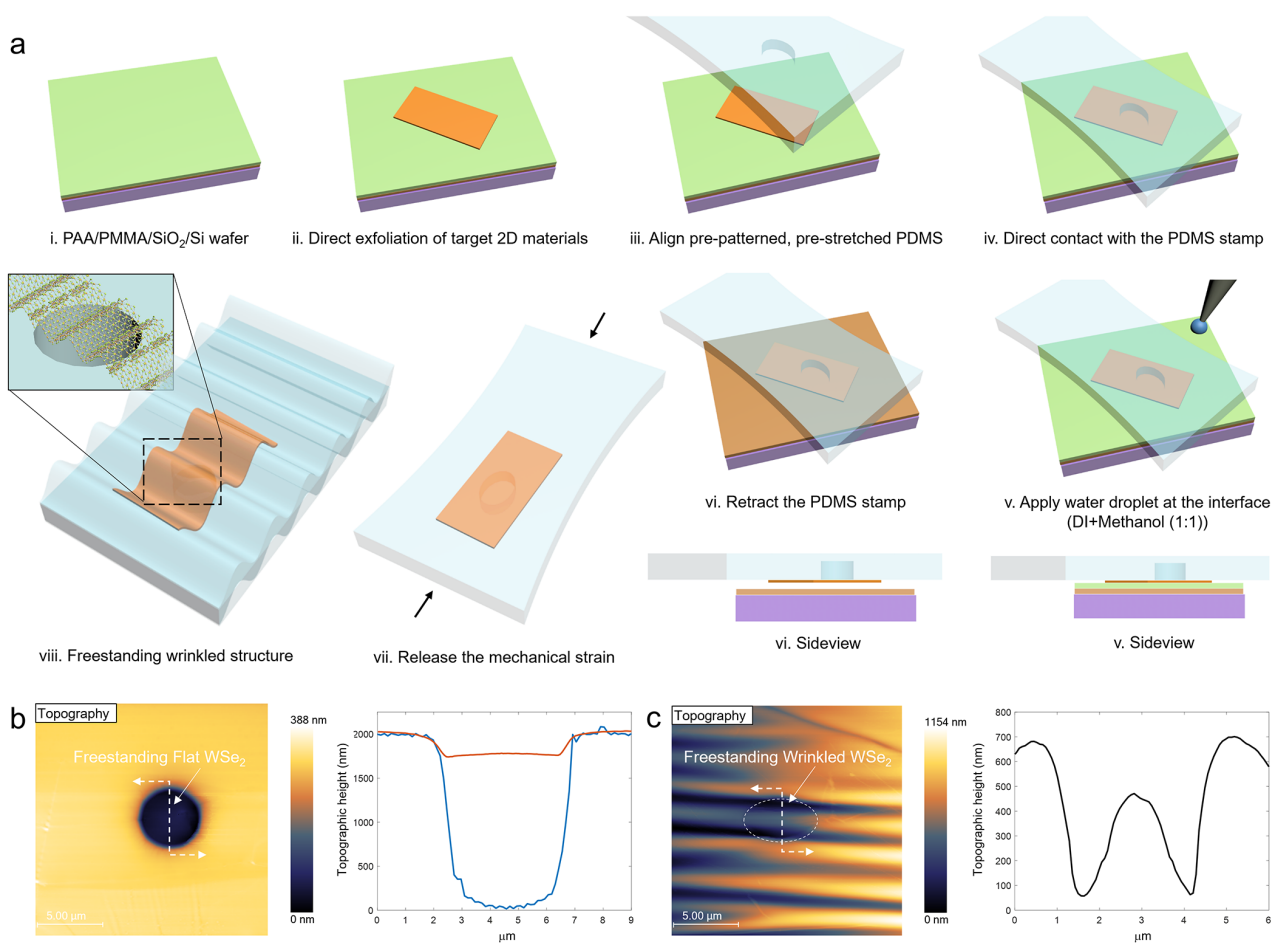
Fabrication of freestanding structures of 2D materials. (a) Schematic illustrations of fabrication process to create freestanding wrinkled structure of 2D materials via a combination of droplets-assisted transfer technique and mechanical prestrain release mechanism. (b) Topographic height image (left) and line profiles (right) of flat freestanding monolayer WSe_2_ (red) transferred over a 4 μm diameter circular cavity in a PDMS substrate (blue). (c) Topographic height image (left) and a line profile (right) of wrinkled freestanding monolayer WSe_2_ over a 4 μm diameter circular cavity after the release of the mechanical strain.

Similarly, to create freestanding wrinkle structures, we used a prestretched (∼120% uniaxial tension), cavity-patterned PDMS substrate with a mild oxygen plasma treatment to create the stiff skin layer. This stiff skin layer on the PDMS was used to guide and control the out-of-plane deformation of both substrate-supported conformal wrinkling and freestanding wrinkling of the transferred 2D materials when the substrate was contracted after the release of the prestretched PDMS.^6^ In addition, the stiff skin layer enables an increased strain transfer by reducing the Young’s modulus mismatch between the PDMS and the 2D TMDC layer.^9^ A thin layer of Teflon (<10 nm) was subsequently coated on the stretched skin-layer/PDMS to make the surface more hydrophobic, promoting a much smoother liquid extraction meniscus at the contact interface during the stamp retraction step (Figure 1a-vi). Then, once the target layer was transferred over the predefined cavities in the prestretched PDMS in the manner described above, we gently released the mechanical prestrain to create a controlled freestanding wrinkle (Figures 1a-viii and S2). The depth of a prepatterned 4 μm diameter hole in PDMS without any 2D materials on top was measured to be about 2 μm (Figure 1b, blue). The fabricated freestanding flat 2D material showed about a 300 nm fall from the surface while maintaining a flat surface (Figure 1b, red), clearly indicating a freestanding structure. On the other hand, topographic height scans of the fabricated freestanding wrinkled 2D materials (Figure 1c) showed periodic wrinkles having a wavelength of 2.3 μm and a height of 360 nm.

Following realization of freestanding structures, we investigated how optical signals are modulated in the absence of an underlying supporting substrate using photoinduced force microscopy to characterize near-field light–matter interaction. By implementing the first and second mechanical resonance modes of atomic force microscope tip operation during the photoinduced force measurement,^20,21^ we simultaneously imaged both topography and photoinduced force without crosstalk as shown in Figure 2 (see Supplementary Note 1). The line profiles taken at the center of the fabricated hole without 2D materials for both topographic height (Figure 2a top) and PiFM (Figure 2a middle) showed that there was no noticeable contrast in photoinduced force over the hole cavity and the surrounding areas (Figure 2a, bottom). However, we observed a strong contrast in the measured photoinduced force when there was a freestanding 2D material suspended over the hole cavity. The photoinduced force signal measured in freestanding monolayer WSe_2_ showed 21.85% increase relative to that in the supported WSe_2_ (Figure 2b). Freestanding monolayer MoS_2_ showed 21.81% increased photoinduced force compared to that in the supported MoS_2_ (Figure 2c), and freestanding heterobilayer MoS_2_/WSe_2_ showed 26.46% increased photoinduced force compared to that in the supported MoS_2_/WSe_2_ (Figure 2d). The negligible contrast in photoinduced force over the bare hole cavity implies that there is no strong polarization from the PDMS substrate at the fixed wavelength of 658 nm visible light excitation. In contrast, the enhanced photoinduced force signals in freestanding structure are attributed to the removal of the surrounding dielectric environment (eq 1 in Supplementary Note 1). It has been reported that photoinduced force measurement is sensitive to small variations in the dielectric environment of the surface.^20,22^ We also observed similar optical response enhancements in freestanding 2D TMDCs with the far-field optical characterizations where we observed increased photoluminescent intensity over the freestanding area compared to that over the flat area (Figure S1 and Supplementary Note 2). Overall our results suggest that freestanding structures enable probing the intrinsic optically driven photoinduced forces in 2D materials whereas we observed suppressed photoinduced force with the presence of underlying substrate.

**Figure 2:**
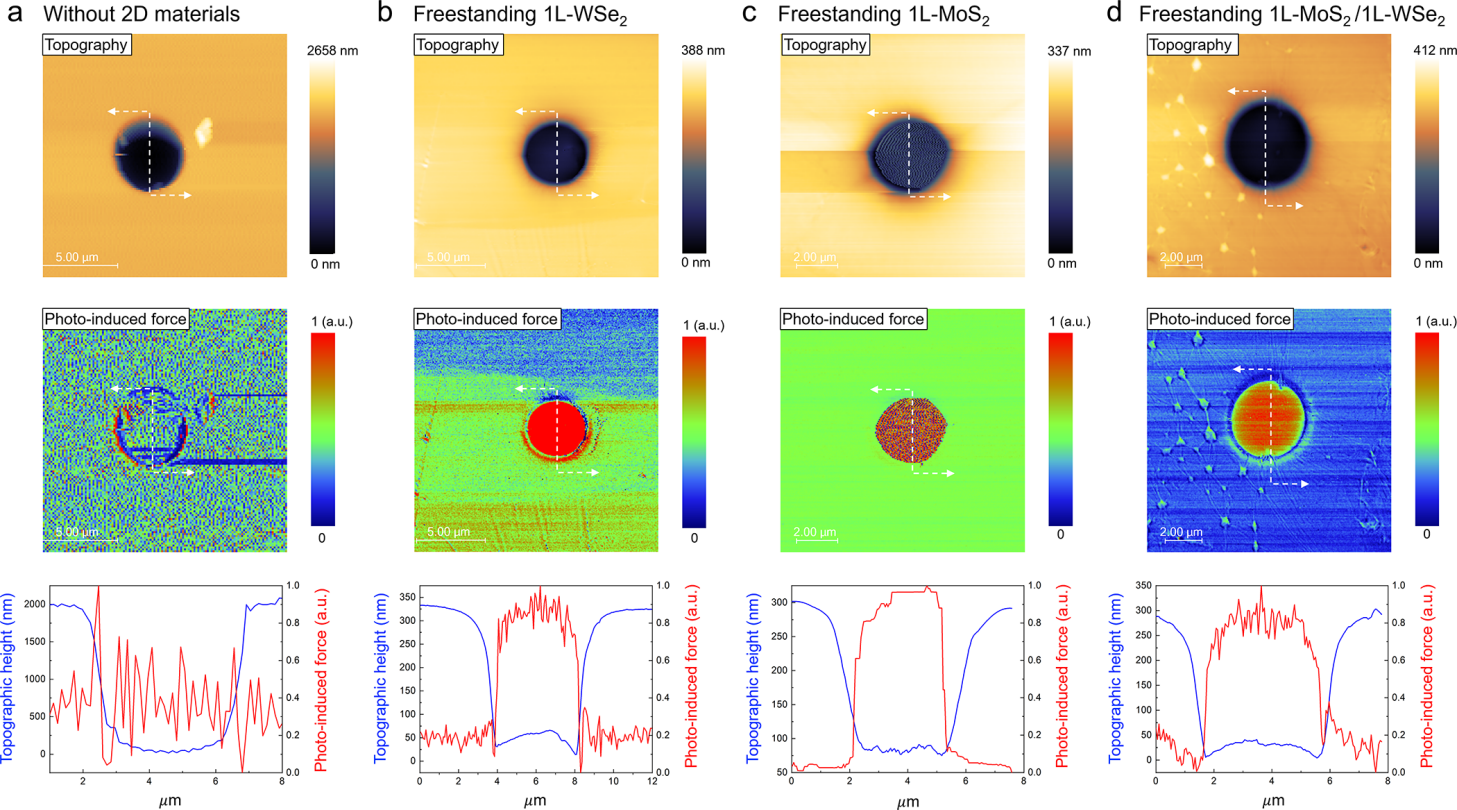
Near-field optical characterizations of the fabricated freestanding flat 2D materials via photoinduced force microscopy. Topographic height scans (top), photoinduced force mappings (middle), and correlation plots (bottom) between topographic height and the measured photoinduced force (normalized) along the white dotted lines of (a) a reference bare PDMS substrate without any deposited 2D materials, (b) a freestanding monolayer WSe_2_, (c) a freestanding monolayer MoS_2_, and (d) a freestanding vertical heterostructure of MoS_2_/WSe_2_ suspended over 4 μm diameter circular cavities in PDMS substrates.

To study how strain can influence the light–matter interaction in monolayer 2D TMDCs, we performed PiFM characterizations of freestanding wrinkled structure of WSe_2_ (Figures 3a and S2). Without any light excitation (Figure 3b), there was a negligible PiFM signal detected over the wrinkled freestanding WSe_2_. However, with the light illumination at 658 nm, we observed the substantial photoinduced force over the wrinkled freestanding WSe_2_ area (Figure 3c). Remarkably, we observed spatially localized photoinduced forces near the apex of the upward-bent wrinkle area (Figure 3d,e). Figure 3f shows that the spatially localized photoinduced force measured at the apex of freestanding wrinkled WSe_2_ was about 151.1% relative to the photoinduced force measured at the valley of freestanding wrinkles (i.e., downward-bent wrinkles). The localization of photoinduced force at the apex can be seen by the spatial confinement of the signal which was substantially narrower, whereas the photoinduced force measured in unstrained freestanding WSe_2_ (Figure 2b) was spatially uniform.

**Figure 3:**
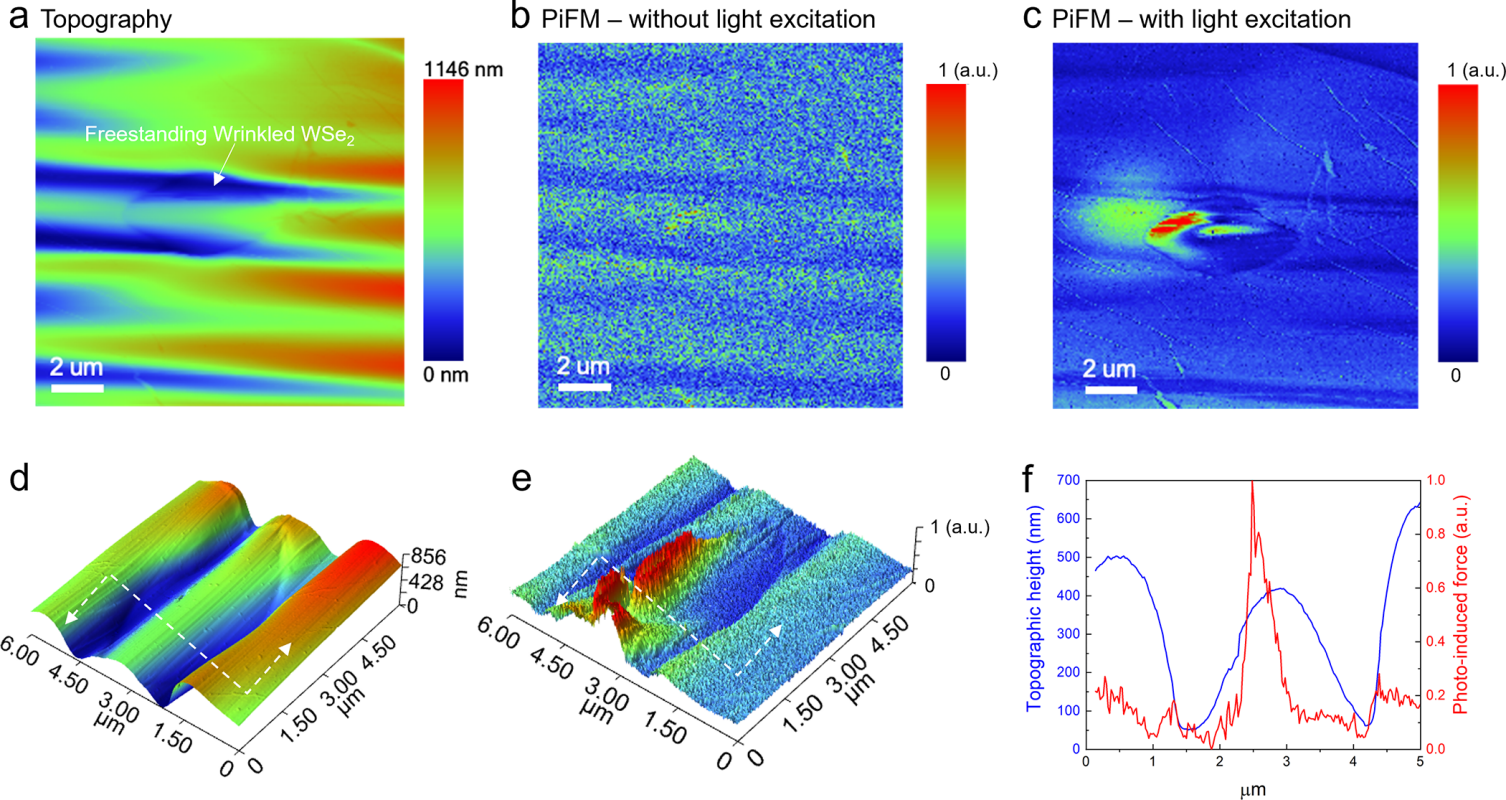
Near-field optical characterizations of the fabricated freestanding wrinkled structure of monolayer WSe_2_ via photoinduced force microscopy. (a) Topographic height of the fabricated freestanding wrinkled WSe_2_ suspended over a 4 μm diameter circular cavity. (b) Photoinduced force mapping without light excitation. (c) Photoinduced force mapping with light excitation at 658 nm. (d) Magnified 3D images of topographic height and (e) photoinduced force measurements over the freestanding wrinkled WSe_2_. The white dotted line indicates the location of line profile taken over the circular cavity. (f) Comparison plot between the measured line profiles of topographic height and normalized photoinduced force via photoinduced force microscopy. A spatially localized photoinduced force was measured at the apex of the fabricated wrinkled structure of monolayer WSe_2_.

Deformation of freestanding 2D materials, such as wrinkling, can induce both structural changes and strain effects, leading to the modulation of optical responses. From the perspective of structural effects on optical responses, given that there is no refractive index contrast (only air) between the medium above and below the suspended 2D TMDCs, we can expect the structural changes leading to the modulation of the absorption in 2D TMDCs owing to light capture in an index mismatched substrate will be negligible.^15^ In contrast, strain effects resulting from the material deformation (i.e., wrinkling) can play a critical role in spatially modulating the optically driven photoinduced force in freestanding wrinkled 2D TMDCs due to direct changes in lattice structures and thus the resultant optical characteristics.

Wrinkling 2D TMDCs in the out-of-plane direction can lead to nonuniform deformation and the corresponding out-of-plane strain gradient through the thickness of the material. The out-of-plane strain gradient would induce an electrical polarization along the thickness direction, which is termed flexoelectric polarization, generating a dipole moment.^23^ Extensive first-principles calculations on the coupling effects between the strain gradient and charge polarization in 2D TMDCs suggested that wrinkling can cause substantial flexoelectricity in TMDC monolayers where the maximum dipole moments and strain gradients increased with the decreased wavelength,^24^ and the out-of-plane polarization is proportional to the curvature.^25^ As shown in Figure 3f, we observed an increase in the photoinduced force at the apex of the wrinkle, specifically where the topography showed a larger curvature (or a smaller radius of curvature). This indicates that refining the wrinkle geometry to reduce its wavelength and increase its amplitude (thus enhancing the strain gradient) would be an optimal strategy to maximize the photoinduced force when utilizing wrinkled structures.

We hypothesize that the strain-gradient-induced dipole moments may interfere either constructively or destructively in the photoinduced force measurement of the wrinkled TMDCs depending on the curvature directions. Atomic dipole moments in the wrinkled TMDCs would possess positive or negative dipole moments depending on the polarization directions where dipole moment direction would be opposite between the wrinkle apex and valley.^24,25^ Such generated flexoelectric dipole moments are expected to be added to the net dipole moment as built-in dipole moments^26^ in freestanding wrinkled WSe_2_, in addition to the optically induced dipole moment. Out-of-plane electric polarization resulting from the strain-gradient effect will directly affect the localized photoinduced force which is proportional to the out-of-plane component of incident field^20^ (*E*_Z_ in eq 1 of Supplementary Note 1). Photoinduced force has been reported to be sensitive to the local electric fields of polarizable materials.^22,27^ Accordingly, we can expect that the generated flexoelectric polarizations having different directions at the wrinkle apexes and valleys will act as built-in dipole moments in the out-of-plane direction adding either constructively or destructively to the optically driven photoinduced force in the wrinkled freestanding monolayer. More specifically, at the apex of wrinkle where the freestanding monolayer WSe_2_ bent upward (i.e., a positive curvature), the bending-induced strain gradient generated through the thickness direction will induce vertical flexoelectric polarizations with the direction from the bottom to the top surface of WSe_2_ generating a positive dipole moment,^24^ increasing the net dipole moment with the optically driven dipole moments and thus increasing measured photoinduced force. In contrast, the bending-induced strain gradient at the valley of wrinkle will generate vertical flexoelectric polarization with the direction from the top to the bottom surface of WSe_2_ generating a negative dipole moment destructively contributing to the net dipole moment during photoinduced force measurement.

We also hypothesize that the strain-gradient effects will be more dominant in the freestanding wrinkled structure compared with the substrate-supported wrinkled structure. This is due to the strain relaxation between the deposited 2D layer and the underlying substrate as substantially different elastic properties and Poisson’s ratios^28,29^ or surface tractions^30^ between the 2D layer and the underlying substrate may result in reduced strain effects at the bottom surface of the 2D layer, weakening the overall strain gradient generated in the overlaid 2D layer relative to the freestanding 2D layer and thus suppressing flexoelectric polarizations.

To validate our hypothesis on the strain-gradient effects on the spatial tuning of photoinduced force, we studied correlations between the topography-driven curvature and the measured photoinduced force profiles. First, we modeled a freestanding 2D material based on nonlinear von Karman elastic plate theory which is valid for the freestanding 2D material where thickness is much smaller than any other characteristic length in the system such as wavelength, or lateral dimensions of width and length with the assumption of small strains, moderate rotations, and no changes in its thickness. The curvature can be expressed as a function of the first and second derivatives of the wrinkle geometry as , where *w* is the out-of-plane deflection and *t* is the thickness of 2D materials. Here the curvature is defined as positive for the wrinkle bent upward (i.e., apex) and as negative for the wrinkle bent downward (i.e., valley). In the freestanding wrinkled monolayer WSe_2_ (Figure 4a, yellow area), the topography-driven curvature parameter (blue line in Figure 4a) shows a positive correlation with the measured photoinduced force (red line in Figure 4a) where the photoinduced force is highly localized/increased around the positive curvatures with the maximum photoinduced force matching with the maximum curvature. In contrast, even though the curvature (i.e., strain gradient) of substrate-supported wrinkled monolayer WSe_2_ areas was slightly larger than that of the freestanding wrinkles, there was negligible modulation or correlation between the topography-driven curvature parameters and the measured photoinduced force. The enhancement of near-field optical response in the apex of freestanding wrinkled WSe_2_ was about 330% relative to that in the apex of substrate-supported wrinkled WSe_2_. To further substantiate our hypothesis of the out-of-plane flexoelectric effects on the spatial modulation of the photoinduced force, we measured photoinduced force of nanoscale transfer-induced wrinkle bent downward (i.e., valley) having a width of about 13.7 nm and an amplitude of 5.9 nm (Figures 4b and S3). We observed reduced photoinduced force (red line in Figure 4b) over the transfer-induced wrinkle having a negative curvature (blue line in Figure 4b), supporting the positive correlation between the flexoelectric effect and the photoinduced force generation.

**Figure 4:**
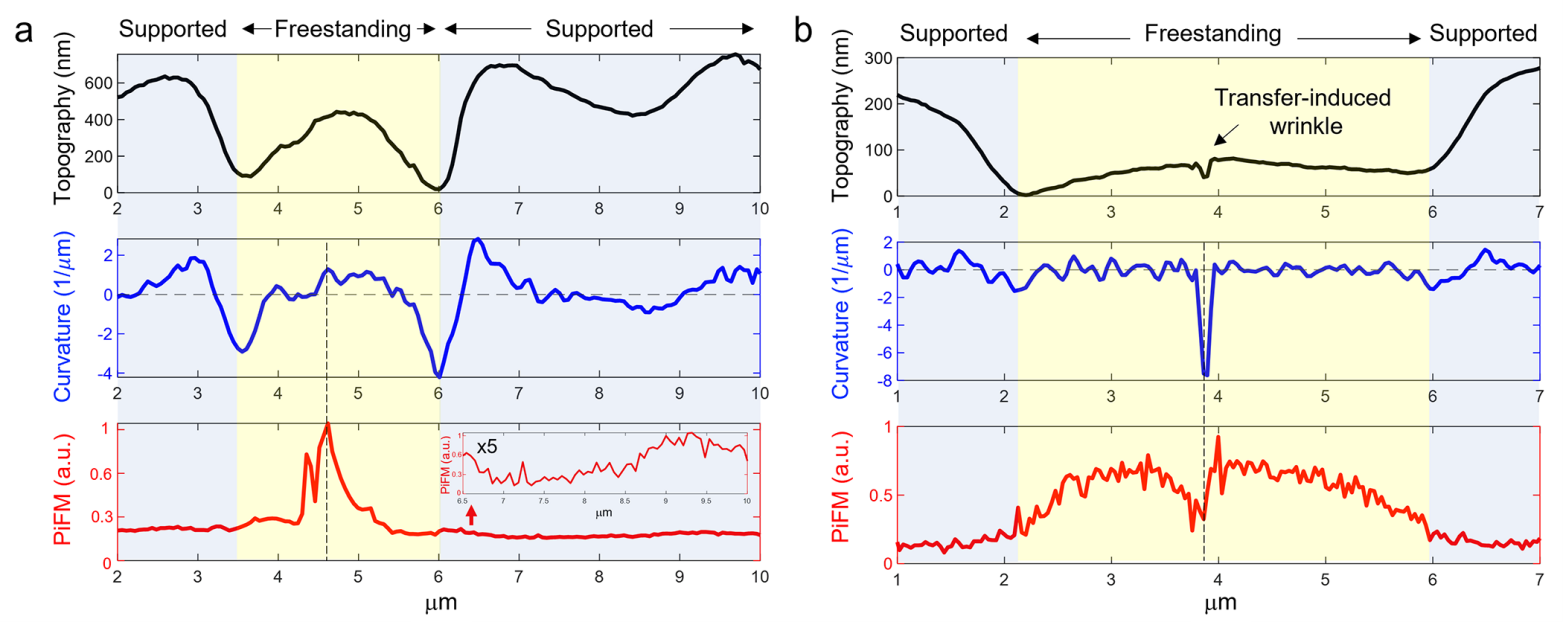
Topography-driven strain gradient correlation with photoinduced force. (a) Correlation plots between measured topography (black), the estimated strain gradient (blue), and the measured normalized photoinduced force (red) over the freestanding wrinkled monolayer WSe_2_ and the substrate-supported wrinkled monolayer WSe_2_ areas. The inset shows about a 5 times magnified measured photoinduced force profile at the supported area. (b) Correlation plots over the freestanding flat monolayer WSe_2_ with a transfer-induced wrinkle.

We also investigated in-plane strain effects on photoinduced forces (Figures S4–S6). In our freestanding wrinkles, we observed only in-plane strains due to the edge-pinning around the cavity periphery and no noticeable in-plane strain over the freestanding area, confirmed by both photoluminescence spectroscopy and topography-driven strain analysis. We did not find any strong correlations between topography-driven in-plane strain and the measured photoinduced force, and thus we attribute spatial modulation of light–matter interaction in the freestanding wrinkled structure to strain gradient, not in-plane strain (see Supplementary Note 3 for in-plane strain effect studies). Overall, our results imply that we can concentrate strain-gradient effects within 2D materials by eliminating the underlying substrate and therefore substantially enhance the out-of-plane polarization and dipole moment in the freestanding wrinkled 2D materials.

In summary, we report strain gradient effects on the optical characteristics of freestanding structures of 2D TMDCs without the effect of substrates or surrounding dielectric environments that have been reported to be detrimental to the optical properties of atomically thin 2D TMDCs. We observed substantially enhanced light–matter interactions in a freestanding structure of various 2D TMDCs including monolayer and heterobilayer structures measured via photoinduced force microscopy. Our findings further suggest that the photoinduced force in freestanding wrinkled 2D materials can be spatially modulated by the out-of-plane strain gradient-induced flexoelectric polarization without any interfacial strain disturbances from the underlying substrate. Our observation of the out-of-plane optical interaction with strain-gradient induced flexoelectric effects offers new opportunities for an additional degree of freedom to address the fundamental limitation of appalling angular performance in 2D materials due to negligible out-of-plane optical response.^31^ More broadly, our approach of deforming 2D materials without a supporting substrate offers a new route toward the realization of novel strain-tunable freestanding 2D material-based optoelectronic devices.

## Supporting Information Available

Droplets-assisted transfer, freestanding 2D materials, freestanding wrinkled structures, spatial modulation of photoinduced force; additional experimental data (PDF)
