# Supplementary material for: Spatial Tuning of Light–Matter Interaction via Strain-Gradient-Induced Polarization in Freestanding Wrinkled 2D Materials
Source: Nano Lett. 2023 Oct 5;23(20):9340–6. doi: 10.1021/acs.nanolett.3c02550 (PMC10603806; doi:10.1021/acs.nanolett.3c02550)
Supplement: Supplementary file 1 — nl3c02550_si_001.pdf [file nl3c02550_si_001.pdf]

Supporting Information

Spatial tuning of light-matter interaction via strain-gradient induced polarization in freestanding wrinkled 2D materials

Chullhee Cho[1,2], Zhichao Zhang[1], Jin Myung Kim[3], Peiwen J. Ma[3], Md Farhadul Haque[1], Peter Snapp[1,4], SungWoo Nam*[3,5]

[1]Department of Mechanical Science and Engineering, University of Illinois Urbana-Champaign, Illinois 61801, USA

[2]Cryogenics and Fluids Branch, NASA Goddard Space Flight Center, Maryland 20771, USA

[3]Department of Mechanical and Aerospace Engineering, University of California Irvine, California 92697, USA

[4]Detectors Systems Branch, NASA Goddard Space Flight Center, Maryland 20771, USA

[5]Department of Materials Science Engineering, University of California Irvine, California 92697, USA

*E-mail: sungwoo.nam@uci.edu

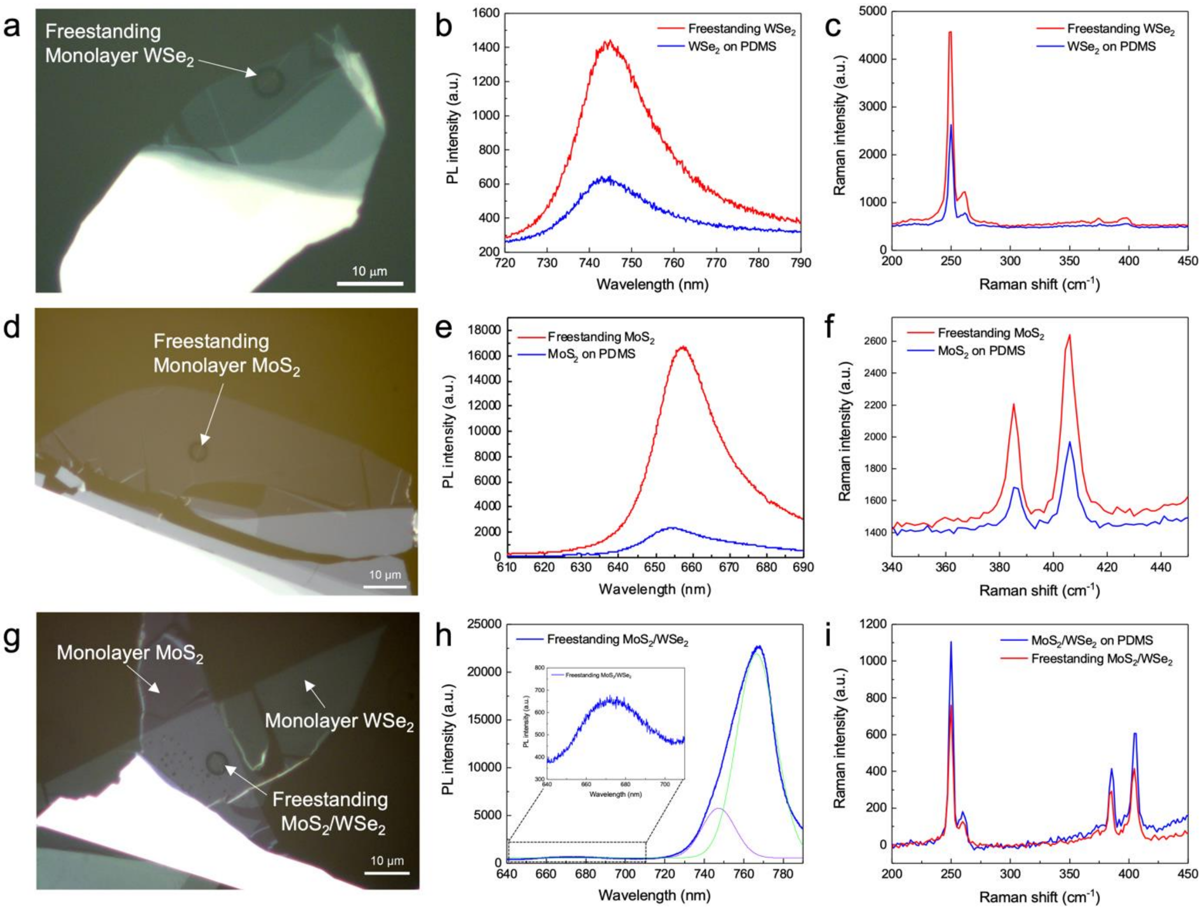

Figure S1. Far-field optical characterization of the fabricated freestanding 2D materials via photoluminescence (PL) and Raman spectroscopy. **a**, An optical microscope image of the fabricated freestanding monolayer WSe_2 suspended over 4 μm-diameter circular cavity on a flat polydimethylsiloxane (PDMS). **b**, PL spectra and **c**, Raman spectra of freestanding WSe_2 (red) and WSe_2 on PDMS substrate (blue). **d**, An optical microscope image of the fabricated freestanding monolayer MoS_2. **e**, PL spectra and **f**, Raman spectra of freestanding MoS_2 (red) and MoS_2 on PDMS substrate (blue). **g**, An optical microscope image of the fabricated freestanding heterostructure of MoS_2/WSe_2. **h**, PL spectrum of freestanding MoS_2/WSe_2 heterostructure. The blue line is the measured PL spectrum and an inset shows a magnified PL peak at the monolayer MoS_2 bandgap energy. Multiple PL peak fitting of the measured PL spectrum shows a peak at the monolayer WSe_2 bandgap energy (violet) and a peak at the momentum space indirect interlayer exciton bandgap energy (green). **i**, Raman spectra of freestanding MoS_2/WSe_2 (red) and MoS_2/WSe_2 on PDMS substrate (blue).

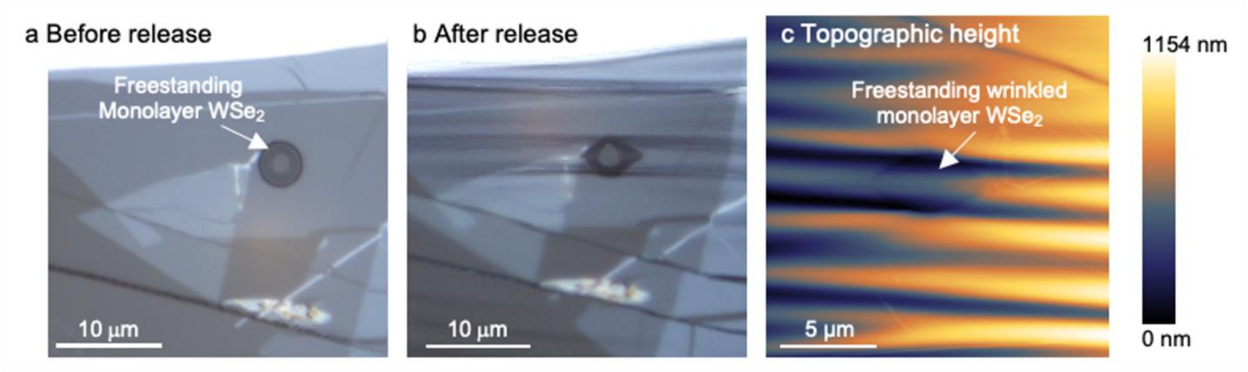

Figure S2. Characterizations of the fabricated freestanding wrinkled structure of monolayer WSe_2. Optical microscope images of the fabricated freestanding monolayer WSe_2 suspended over a 4 μm-diameter circular cavity **a**, before and **b**, after the release of the pre-stretched PDMS substrate. **c**, Atomic force microscopy (AFM) topographic height scan of the fabricated freestanding wrinkled structure of monolayer WSe_2.

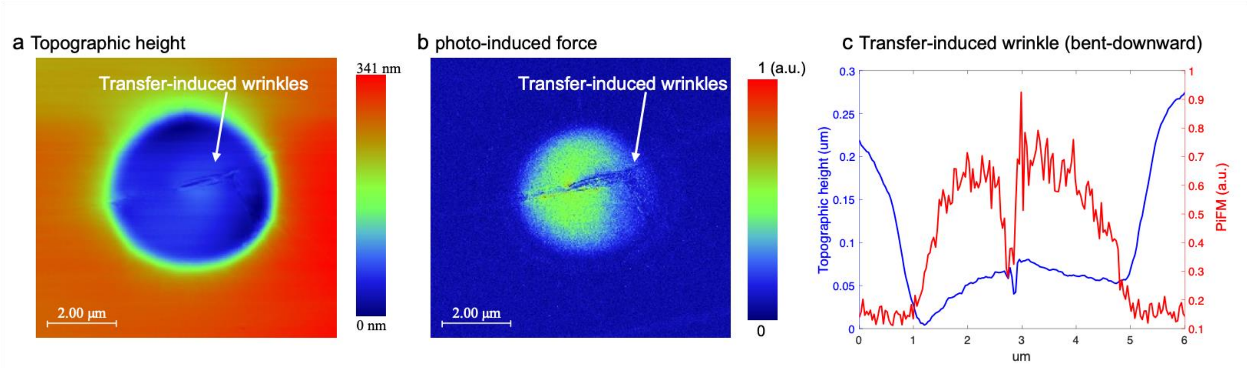

Figure S3. Near-field optical characterizations of the fabricated freestanding monolayer WSe_2 with transfer-induced wrinkles via photo-induced force microscopy. **a**, Topographic height and **b**, photo-induced force mappings of the fabricated freestanding monolayer WSe_2 with transfer-induced wrinkles across the center of the 4 μm-diameter circular cavity. **c**, A comparison plot between the measured topographic height and normalized photo-induced force over a transfer-induced wrinkle bent downward.

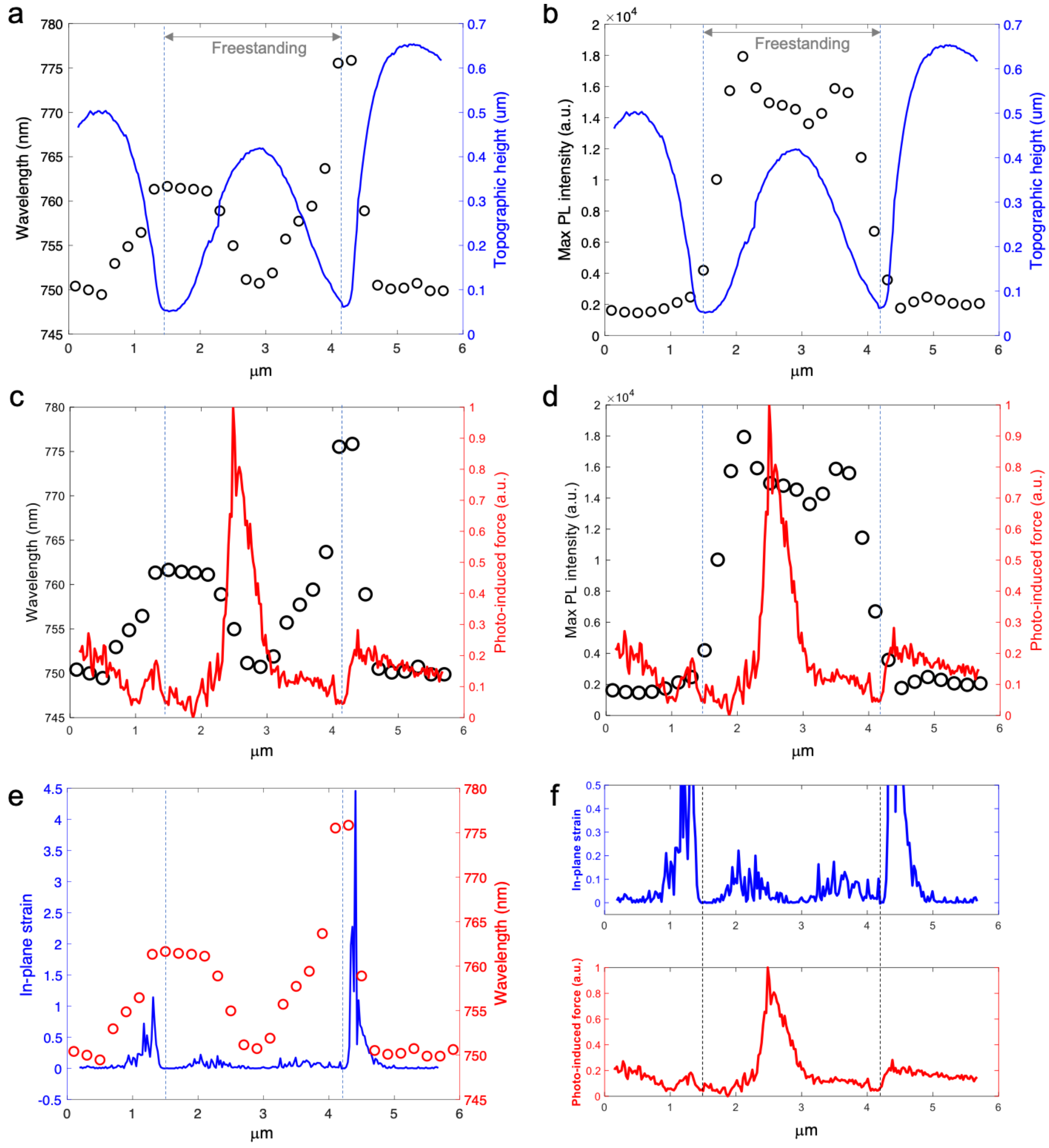

Figure S4. Strain correlation plots between spatially resolved PL spectroscopy, photo-induced force microscopy, and topography-driven in-plan strain. **a**, A correlation plot between the PL peak wavelength and topography. **b**, A correlation plot between the maximum PL intensity and topography. **c**, A correlation plot between the PL peak wavelength and photo-induced force. **d,** A correlation plot between the maximum PL peak intensity and photo-induced force. **e**, A correlation plot between the estimated in-plane strain (blue) and the measured PL peak wavelength shift (red). **f**, A correlation plot between the estimated in-plane strain (blue) and the measured photo-induced force (red).

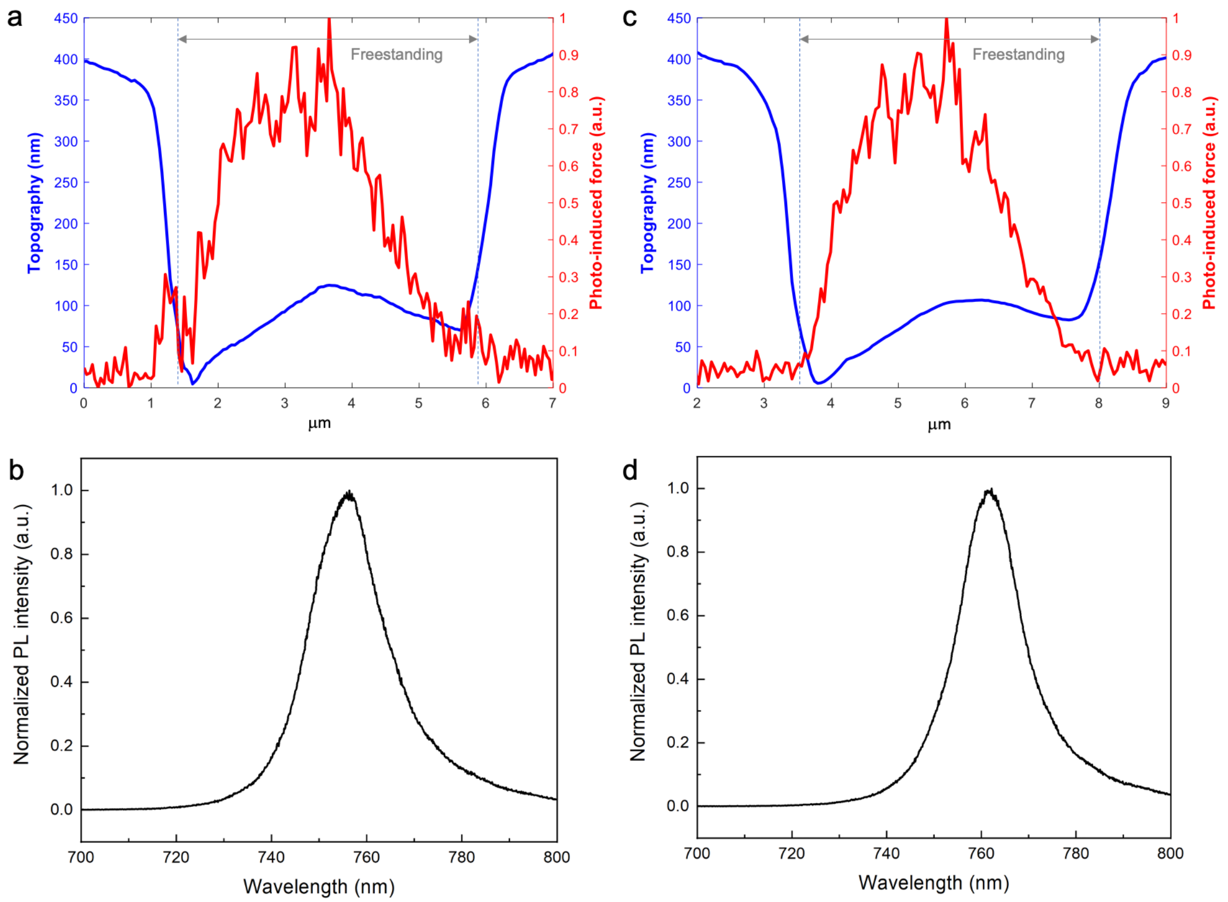

Figure S5. In-plane strain analysis of freestanding, flat monolayer WSe_2 under different levels of in-plane strain measured via photo-induced force microscopy and photoluminescence spectroscopy. **a**, A comparison plot between measured topography and photo-induced force over a flat freestanding monolayer WSe_2, **b**, Photoluminescence (PL) spectral measurement of the flat freestanding monolayer WSe_2, **c,** A comparison plot between measured topography and photo-induced force over a flat freestanding monolayer WSe_2 under slight tension, **d**, PL spectral measurement of the flat freestanding WSe_2 under slight tension.

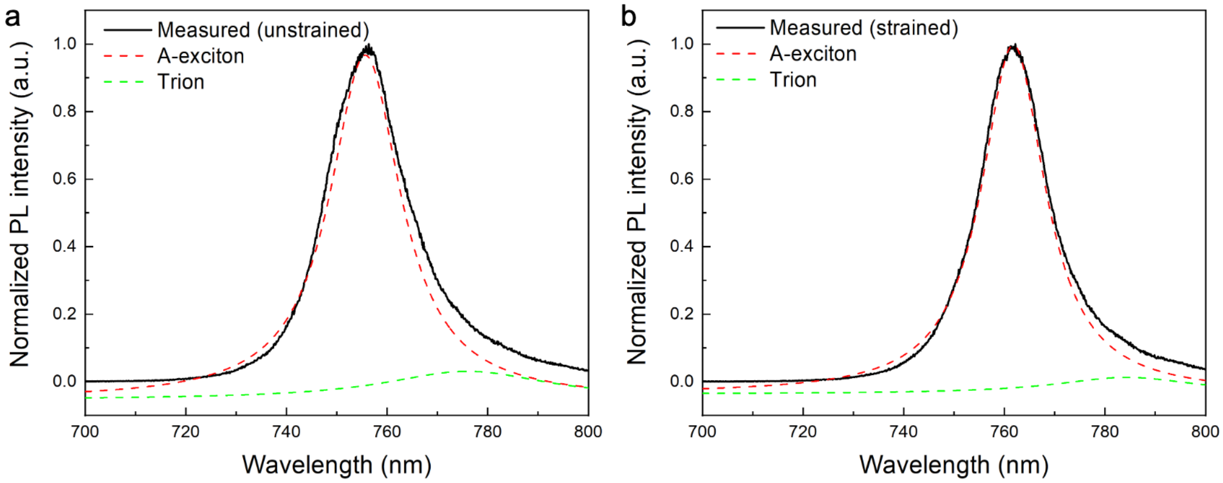

Figure S6. Photoluminescence spectra measurement with peak fitting using Lorentzian function over **a**, A flat (unstrained) freestanding monolayer WSe_2, and **b**, A strained freestanding monolayer WSe_2.

Supplementary Note 1: Working mechanism of photo-induced force measurement

Photo-induced force microscopy (PiFM) measures the near-field optical interaction based on sample polarizability, primarily the induced dipole-dipole interaction (attraction force) between the optically driven dipole and the dipole in metal-coated atomic force microscopy (AFM) tip when illuminated with a coherent light source, through the deflection of the AFM cantilever tip, allowing for a true near-field technique[1,2]. Here, the metal-coated AFM tip (in our case gold-coated tip) enables large values of tip polarization and thus offers the measurement of electric dipole-dipole interaction forces. The local polarization of the sample by the near-field excitation results in highly localized forces exerting between the tip and sample. Such near-field detection minimizes far-field backgrounds contribution of scattered photons surrounding the tip apex which have been one of the general challenges for tip-enhanced measurements[1]. When the light illuminates at the tip-sample junction, it induces dipoles in the tip and the sample mutually interacting with each other generating an attractive Coulombic force. The time-averaged localized photo-induced force (F) can be described with the dipole approximation of point dipoles (i.e., polarizable spheres) assuming a non-varying spatial phase of the electric fields:

$$< F > \propto -\frac{1}{z^4} Re\{\alpha_s \alpha_t\} |E_z|^2 \text{ (refs }^{1,3}\text{)} \quad (1)$$

where α_s and α_t are the polarizabilities of sample and the tip, respectively, z is the distance from the center of the dipole to surface, and E_z is out-of-plane components of the incident field. As shown in Eq. 1, both E_z and $\frac{1}{z^4}$ dependence result in the highly localized optical force detection with the high spatial resolution[1]. In our experiments, the scan speed was 0.5 lines per second at 256 x 256 pixels. We note that we used a low scan rate of 0.5 lines per second to ensure an identical tip-sample distance in both trace and retrace scans and adequate integration time for the lock-in amplifier to minimize any undesirable tip-sample perturbations. We used an excitation light source having a fixed wavelength at 658 nm with a time constant of 10 ms and a 50% laser duty cycle.

Supplementary Note 1 (continued):

While PiFM is operated in tapping mode similar to scattering near-field scanning optical microscopy (s-SNOM), there is no need for higher order multiple demodulations allowing minimized compromised signals unlike s-SNOM. Moreover, PiFM offers detection and mechanical-imaging of nanoscale light-matter interaction over a wide range of wavelengths from visible to infrared without any far-field contribution. Optically induced forces measured by PiFM have recently attracted fundamental importance in applications such as optical trapping[4], chemical imaging[1,5], and nanoscale spectroscopy[2]. In general, the overall measured photo-induced force includes thermally induced expansion force in addition to the electromagnetic gradient force[6]. In our experiment, however, we used atomically-thin monolayer of semiconducting WSe_2 (thickness of 0.67 nm) under visible light excitation (658 nm wavelength) where the photo-thermal expansion force is expected to be very small due to 1) its ultra-thin thickness having a very small thermal mass and its high thermal conductivity (~49 W/m-K) contributing to effectively dissipating heat and preventing any heat accumulation to raise thermal expansion[7], and 2) a low light absorbance of only about 5.8% in the wavelength range from 450 to 800 nm for both suspended and supported samples[7,8]. Thermal expansion force, in general, is found to be dominant for thicker films (thickness > 100 nm) under mid-infrared radiation, whereas the gradient force is the dominant force for nanosheet/particles and under visible light excitation[6]. As a result, we expect the heating effect contributing to the enhanced photo-induced force will be negligible.

Supplementary Note 2: Far-field optical characterization of freestanding flat 2D materials

To investigate substrate decoupling effect on the optical characteristics of freestanding 2D materials, we start with comparing non-destructive far-field optical characterizations of photoluminescence (PL) and Raman spectroscopy on the freestanding WSe_2 (Fig. S1a), MoS_2 (Fig. S1d), and MoS_2/WSe_2 heterostructure (Fig. S1g) to those of their counterparts on PDMS substrates. The fabricated freestanding monolayers and heterostructure show substantial increases in PL intensities versus counterparts on PDMS substrate under

the same incident power (Fig. S1b,e,h). In the particular case of freestanding MoS_2/WSe_2 heterostructure (Fig. S1h), the enhanced PL signal enables to clearly show the PL peak of momentum-indirect ($\Gamma - K$) interlayer excitonic resonance at 1.58 eV, where the measured PL spectra show substantially quenched A excitons of MoS_2 (blue spectrum in the inset of Fig. S1h) and WSe_2 (violet) with the dominant interlayer exciton peak (green).

Next, we compared Raman spectra between the supported and freestanding monolayers and heterostructures. Raman spectroscopy characterizes lattice vibration modes that can provide strain, doping, and the number of layer configurations. Similar to PL spectra comparison, we also observed that freestanding 2D materials can enhance both the intensity and resolution of their Raman signals. Raman intensities of the dominant first-order Raman modes of in-plane E_{2g} and out-of-plane A_{1g} in freestanding monolayer WSe_2 (Fig. S1c) and MoS_2 (Fig. S1f) were stronger compared to those in their supported counterparts on PDMS substrate. In the particular case of freestanding WSe_2 in Figure S1c, we observed the enhanced sensitivity towards phonon modes ranging from 300 to 400 cm^{-1} that are associated with second-order processes involving two phonons within the interior of the Brillouin zone (i.e., second-order overtone Raman modes), which are relatively weak to be defined in the supported WSe_2 monolayer. In cases of freestanding MoS_2 and MoS_2/WSe_2 heterostructure, we observed that both in-plane (E_{2g}) and out-of-plane (A_{1g}) modes of MoS_2 were softened compared to the supported counterparts. Both red-shifted E_{2g} (~ - 0.7 cm^{-1}) and A_{1g} (~ - 0.2 cm^{-1}) Raman peaks indicate that the freestanding area is slightly under tension and possesses the reduced out-of-plane interaction with the underlying substrate.

We attribute the enhanced sensitivities and intensities of PL and Raman to the changes in the effect of the dielectric environment induced by the underlying substrate (i.e., the substrate decoupling effect) on the local electromagnetic field, where the reduced substrate interaction may enhance the local electromagnetic field and the resultant optical signals. Our results agree well with previous studies where suspended graphene and MoS_2 have shown enhanced Raman and PL signals, which are attributed to the diminishment of detrimental interactions between the materials and substrates, such as nonradiative recombination, charge transfer, and excitonic transition[9,10]. Also, it has been reported that PL efficiencies

can be increased via substrate engineering from supported to suspended monolayers of MoS_2 and WSe_2[11]. Another study showed that the dielectric surroundings around the 2D materials can optically regulate the incoupling and outcoupling of light, yielding a dramatic variation of the emission intensity either enhanced or suppressed depending on the thickness of the underlying substrates[12].

Supplementary Note 3: In-plain strain effects on the photo-induced force in the freestanding wrinkled WSe_2.

We first spatially resolved PL over the wrinkle to probe in-plane strain exerting in the freestanding wrinkled WSe_2. Figure S4 shows comparison plots between the measured PL line scan, the measured wrinkle topography and photo-induced force, and the topography-driven in-plane strain. PL results (Fig. S4a) suggest that the valleys of wrinkles are under tension where they show the maximum PL peak wavelength shift of +24.3 nm, which corresponds to ~51.8 meV shift in resonance PL energy. The apex of the wrinkle is under negligible strain where it shows the PL peak at 750.7 nm (~1.652 eV). According to the previously reported deformation potential of WSe_2 (55 meV/%), the valleys of wrinkles are under tension ranging from 0.44% to 0.96% in-plane strain. We also observed a substantial PL intensity increase over the freestanding wrinkle (Fig. S4b), which is similar to what we observed in the flat freestanding monolayers. When we compare the measured topography of wrinkles and the measured PL intensity, we found that the PL intensities started to increase near the valley of the wrinkle suggesting the in-plane strain probed via PL is due to the edge pinning effects from the periphery of the cavity where there is an abrupt transition from supported to freestanding. We did not find any strong correlation between topography-driven in-plane strain and the measured photo-induced force.

Additionally, we performed photo-induced force microscopy over two different monolayer WSe_2 freestanding samples to explore in-plane strain effect without substantial wrinkle formation. As shown in Figure S5, we did not observe any significant spatial tuning or changes in the measured photo-induced force between the samples even though one flat freestanding WSe_2 was slightly under tension, which was

indicated by the shifted A exciton peak wavelength at about 762 nm (Fig. S5d) compared to the unstrained resonance A exciton peak wavelength of 754 nm (Fig. S5b). We did not find any strong correlation between spatial modulation of photo-induced force and in-plane strain.

When there is an increased in-plane tension exerted in semiconducting 2D materials, we expect to see two dominant effects − electronic bandgap modulation and an increase in the piezoelectric potential. If the in-plane strain-induced bandgap modulation plays a critical role in spatial tuning of photoinduced force, we should have observed an increased photo-induced force with increased tension, because 1) the electronic polarizability of 2D materials is inversely proportional to their optical bandgap energy[13] and thus with the increased tension, which results in lowering the optical bandgap energy, we would expect to see increased electronic polarizability enhancing the photo-induced force. 2) Both experimental[14] and first-principle calculations using density functional theory[15] suggested that a tensile strain increases the optical absorption of WSe_2, which will then lead to an increase in the photo-induced force. Our observation of the reduced photo-induced force with the increased in-plane tension shows a clearly opposite behavior to these effects, ruling out the possible underlying mechanisms of both strain-induced optical bandgap modulation and strain-induced absorption effects. Next, when a static strain is introduced in a strong piezoelectric semiconductor including monolayer WSe_2, the presence of the localized polarization charges can effectively modulate its optical properties through the corresponding strain-induced electrostatic potentials. It has been reported that the strain-induced piezoelectric effects induce various destructive effects on the optical light-matter interactions such as a reduction in exciton recombination and exciton binding energy owing to the piezoelectric polarization field reducing the electron-hole wavefunction overlap[16], and local electronic band tilting[17]. Furthermore, theoretical studies by using transport model including drift and diffusion of electrons and holes on the effect of spontaneous and piezoelectric polarization on the optical characteristics suggested that the influence of piezoelectric polarization on optical properties is more severe than the spontaneous polarization reducing internal quantum efficiency where the best optical performance was predicted without the built-in piezoelectric electrostatic fields[18]. Thus, we hypothesize that the built-in

electrostatic potentials generated by the increased in-plane strain resulting in the increased piezoelectric effects may have a destructive effect on the photo-induced force by disturbing optically-generated dipole in freestanding 2D materials and the incident electromagnetic fields. However, since we observed in-plane strain only around the cavity periphery owing to the edge-pinning effect where also may be influenced by substrate dielectric due to the proximity, it is inconclusive that in-plane strain would have destructive effects on the photo-induced force.

We studied correlations between the topography-driven in-plane strain, and the measured photo-induced force profile. Similar to the curvature studies with the 2D model discussed in the main text, in-plane strain can be expressed as a function of the square of the first derivative of wrinkle geometry $\frac{1}{2}(\frac{\partial w}{\partial x})^2$ and a bending-induced in-plane strain term consisting of the 2nd derivative of the wrinkle geometry $-\frac{1}{2}(\frac{\partial^2 w}{\partial x^2})t$, where w is the out-of-plane deflection, and t is the thickness of 2D materials. Our topography-driven in-plane strain corresponds well to the PL line scan result (Fig. S4e) where it shows a lower bandgap (i.e., a longer wavelength) at the valleys with the increased estimated tension. Again, the in-plane strain did not show any strong correlations with the measured photo-induced force (Fig. S4f) where the maximum photo-induced force was observed at the apex with negligible in-plane strain.

Lastly, we performed photoluminescence spectroscopy with peak fittings using Lorentzian function over freestanding monolayer WSe_2 for both unstrained (A-exciton resonance peak at 754 nm in Fig. S6a) and strained (A-excitonic resonance peak shifted to 762 nm in Fig. S6b) samples. As shown in Figure S6, we did not observe any significant peak broadening near the A-exciton resonance peak of monolayer WSe_2, which is an indication of negligible trion formation and negligible contribution of any trions to the generated photo-induced force.

Supplementary References

(1) Nowak, D.; Morrison, W.; Wickramasinghe, H. K.; Jahng, J.; Potma, E.; Wan, L.; Ruiz, R.; Albrecht, T. R.; Schmidt, K.; Frommer, J.; Sanders, D. P.; Park, S. Nanoscale Chemical Imaging

by Photoinduced Force Microscopy. *Sci. Adv.* **2016**, *2* (3). https://doi.org/10.1126/sciadv.1501571.

(2) Jahng, J.; Potma, E. O.; Lee, E. S. Nanoscale Spectroscopic Origins of Photoinduced Tip-Sample Force in the Midinfrared. *Proc. Natl. Acad. Sci. U. S. A.* **2019**, *116* (52), 26359–26366. https://doi.org/10.1073/pnas.1913729116.

(3) Huang, F.; Tamma, V. A.; Mardy, Z.; Burdett, J.; Wickramasinghe, H. K. Imaging Nanoscale Electromagnetic Near-Field Distributions Using Optical Forces. *Sci. Rep.* **2015**, *5*, 1–12. https://doi.org/10.1038/srep10610.

(4) Grigorenko, A. N.; Roberts, N. W.; Dickinson, M. R.; Zhang, Y. Nanometric Optical Tweezers Based on Nanostructured Substrates. *Nat. Photonics* **2008**, *2* (6), 365–370. https://doi.org/10.1038/nphoton.2008.78.

(5) Stöckle, R. M.; Suh, Y. D.; Deckert, V.; Zenobi, R. Nanoscale Chemical Analysis by Tip-Enhanced Raman Spectroscopy. *Chem. Phys. Lett.* **2000**, *318* (1–3), 131–136. https://doi.org/10.1016/S0009-2614(99)01451-7.

(6) Sifat, A. A.; Jahng, J.; Potma, E. O. Photo-Induced Force Microscopy (PiFM) - Principles and Implementations. *Chem. Soc. Rev.* **2022**, *51* (11), 4208–4222. https://doi.org/10.1039/d2cs00052k.

(7) Li, J.; Chen, Z.; Yang, H.; Yi, Z.; Chen, X.; Yao, W.; Duan, T.; Wu, P.; Li, G.; Yi, Y. Tunable Broadband Solar Energy Absorber Based on Monolayer Transition Metal Dichalcogenides Materials Using Au Nanocubes. *Nanomaterials* **2020**, *10* (2). https://doi.org/10.3390/nano10020257.

(8) Easy, E.; Gao, Y.; Wang, Y.; Yan, D.; Goushehgir, S. M.; Yang, E. H.; Xu, B.; Zhang, X. Experimental and Computational Investigation of Layer-Dependent Thermal Conductivities and Interfacial Thermal Conductance of One- To Three-Layer WSe2. *ACS Appl. Mater. Interfaces* **2021**, *13* (11), 13063–13071. https://doi.org/10.1021/acsami.0c21045.

(9) Robert, C.; Lagarde, D.; Cadiz, F.; Wang, G.; Lassagne, B.; Amand, T.; Balocchi, A.; Renucci, P.; Tongay, S.; Urbaszek, B.; Marie, X. Exciton Radiative Lifetime in Transition Metal Dichalcogenide Monolayers. *Phys. Rev. B* **2016**, *93* (20), 1–10.

https://doi.org/10.1103/PhysRevB.93.205423.

(10) Berciaud, S.; Ryu, S.; Brus, L. E.; Heinz, T. F. Probing the Lntrinsic Properties of Exfoliated Graphene: Raman Spectroscopy of Free-Standing Monolayers. *Nano Lett.* **2009**, *9* (1), 346–352. https://doi.org/10.1021/nl8031444.

(11) Yu, Y.; Yu, Y.; Xu, C.; Cai, Y. Q.; Su, L.; Zhang, Y.; Zhang, Y. W.; Gundogdu, K.; Cao, L. Engineering Substrate Interactions for High Luminescence Efficiency of Transition-Metal Dichalcogenide Monolayers. *Adv. Funct. Mater.* **2016**, *26* (26), 4733–4739. https://doi.org/10.1002/adfm.201600418.

(12) Deng, S.; Berry, V. Wrinkled, Rippled and Crumpled Graphene: An Overview of Formation Mechanism, Electronic Properties, and Applications. *Mater. Today* **2016**, *19* (4), 197–212. https://doi.org/10.1016/j.mattod.2015.10.002.

(13) Tian, T.; Scullion, D.; Hughes, D.; Li, L. H.; Shih, C. J.; Coleman, J.; Chhowalla, M.; Santos, E. J. G. Electronic Polarizability as the Fundamental Variable in the Dielectric Properties of Two-Dimensional Materials. *Nano Lett.* **2020**, *20* (2), 841–851. https://doi.org/10.1021/acs.nanolett.9b02982.

(14) Aslan, O. B.; Deng, M.; Heinz, T. F. Strain Tuning of Excitons in Monolayer WSe_2. *Phys. Rev. B* **2018**, *98* (11), 115308. https://doi.org/10.1103/PhysRevB.98.115308.

(15) Lien, D. H.; Kang, J. S.; Amani, M.; Chen, K.; Tosun, M.; Wang, H. P.; Roy, T.; Eggleston, M. S.; Wu, M. C.; Dubey, M.; Lee, S. C.; He, J. H.; Javey, A. Engineering Light Outcoupling in 2D Materials. *Nano Lett.* **2015**, *15* (2), 1356–1361. https://doi.org/10.1021/nl504632u.

(16) De Palma, A. C.; Cossio, G.; Jones, K.; Quan, J.; Li, X.; Yu, E. T. Strain-Dependent Luminescence and Piezoelectricity in Monolayer Transition Metal Dichalcogenides. *J. Vac. Sci. Technol. B* **2020**, *38* (4), 042205. https://doi.org/10.1116/6.0000251.

(17) Wu, W.; Wang, L.; Yu, R.; Liu, Y.; Wei, S. H.; Hone, J.; Wang, Z. L. Piezophototronic Effect in Single-Atomic-Layer MoS2for Strain-Gated Flexible Optoelectronics. *Adv. Mater.* **2016**, *28* (38), 8463–8468. https://doi.org/10.1002/adma.201602854.

(18) Kuo, Y.-K.; Yen, S.-H.; Tsai, M.-C.; Liou, B.-T. Effect of Spontaneous and Piezoelectric Polarization on the Optical Characteristics of Blue Light-Emitting Diodes. *Seventh Int. Conf. Solid State Light.* **2007**, *6669* (September 2007), 66691I. https://doi.org/10.1117/12.733860.
